# Mesenchymal Stem Cell-Based Therapy for Kidney Disease: A Review of Clinical Evidence

**DOI:** 10.1155/2016/4798639

**Published:** 2016-09-19

**Authors:** Anna Julie Peired, Alessandro Sisti, Paola Romagnani

**Affiliations:** ^1^Excellence Centre for Research, Transfer and High Education for the Development of DE NOVO Therapies (DENOTHE), Florence, Italy; ^2^Department of Biomedical, Experimental and Clinical Sciences, University of Florence, Florence, Italy; ^3^Nephrology Unit, Meyer Children's University Hospital, Florence, Italy

## Abstract

Mesenchymal stem cells form a population of self-renewing, multipotent cells that can be isolated from several tissues. Multiple preclinical studies have demonstrated that the administration of exogenous MSC could prevent renal injury and could promote renal recovery through a series of complex mechanisms, in particular via immunomodulation of the immune system and release of paracrine factors and microvesicles. Due to their therapeutic potentials, MSC are being evaluated as a possible player in treatment of human kidney disease, and an increasing number of clinical trials to assess the safety, feasibility, and efficacy of MSC-based therapy in various kidney diseases have been proposed. In the present review, we will summarize the current knowledge on MSC infusion to treat acute kidney injury, chronic kidney disease, diabetic nephropathy, focal segmental glomerulosclerosis, systemic lupus erythematosus, and kidney transplantation. The data obtained from these clinical trials will provide further insight into safety, feasibility, and efficacy of MSC-based therapy in renal pathologies and allow the design of consensus protocol for clinical purpose.

## 1. Characteristics and Properties of MSC in the Context of Clinical Use

The mesenchymal stem cells (MSC), also called mesenchymal stromal cells, are adherent, fibroblast-like cells capable of self-renewal and multilineage differentiation. They were identified nearly half a century ago from cell cultures of murine bone marrow by Friedstein, who defined them as colony-forming unit fibroblasts [1]. In the bone marrow, MSC constitute 0.01% of all cells and contribute to regulating self-renewal, maturation, and recruitment of hematopoietic stem cells to the vascular compartment through cell-to-cell interaction or secretion of soluble factors such as cytokines, chemokines, and growth factors [2–4]. MSC cultured* in vitro* lack specific and unique markers. Conventionally, they are characterized by (1) the expression of several surface markers such as CD44, CD73, CD90, CD105, CD166, CD271, and Stro-1 together with the absence of CD14, CD34, CD45, and HLA-DR; (2) the capacity to adhere to plastic [2, 3, 5, 6]; (3) the ability to differentiate* in vitro* into mesodermal cell types like osteoblasts, adipocytes, and chondrocytes [6, 7]. Some studies suggest that MSC could transdifferentiate into ectodermal and endodermal lineages, but emerging evidence oppose this view [8–10]. An important aspect to consider for the clinical use of MSC is the fact that the methods used for MSC isolation (enzymatic or nonenzymatic), selection (adherence to plastic, cell sorting, etc.), expansion (culture media, oxygen tension, etc.), and assessment are not yet fully standardized. The ISCT (International Society for Cellular Therapy) proposed in 2006 a series of minimal criteria for isolation and cultivation of the MSC. Further attempts to uniformize the characteristics of MSC used in the clinic have been made since [11–13]. 

Interestingly, the MSC currently used for patient therapy are nonclonal MSC, a more heterogeneous population of cells. In effect, clonal cultures would be more homogeneous and therefore preferable but cannot be expanded into a sufficient number of daughter cells. Therefore, the percentage of stem cells contained in every nonclonal population can vary and must be evaluated independently before clinical use through, for example, colony-forming unit (CFU) assays and the evaluation of the multipotential capacity of CFU [14].* In vitro* expansion is a necessary procedure to obtain a sufficient number of MSC, but the maximum number of* in vitro* passages is mostly nonstandardized. A major risk is that through multiple cycles of replications (25 to 30 population doublings) MSC would give rise to a population of senescent cells [15]. These cells could not only loose MSC properties but also release harmful factors that could damage the surrounding healthy cells. A phase II clinical trial conducted by Le Blanc et al. showed that earlier passage MSC infused into patients with GVHD led to a better disease outcome [16]. Monitoring of senescence, by, for example,* in situ* senescence associated beta galactosidase tests, would allow a better control of the MSC population composition and therefore reduce adverse effects [14, 17].

**Figure 1:**
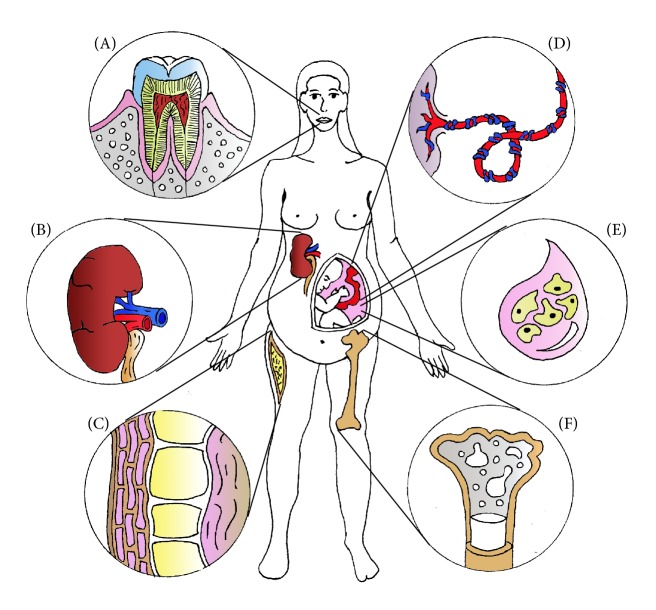
Sources of MSC used in experimental models of renal injury. Preclinical studies have shown that MSC used to treat renal diseases can be isolated from the following tissues: (A) tooth pulp, (B) kidney, (C) adipose tissue, (D) umbilical cord, (E) amniotic fluid, and (F) bone marrow.

**Figure 2:**
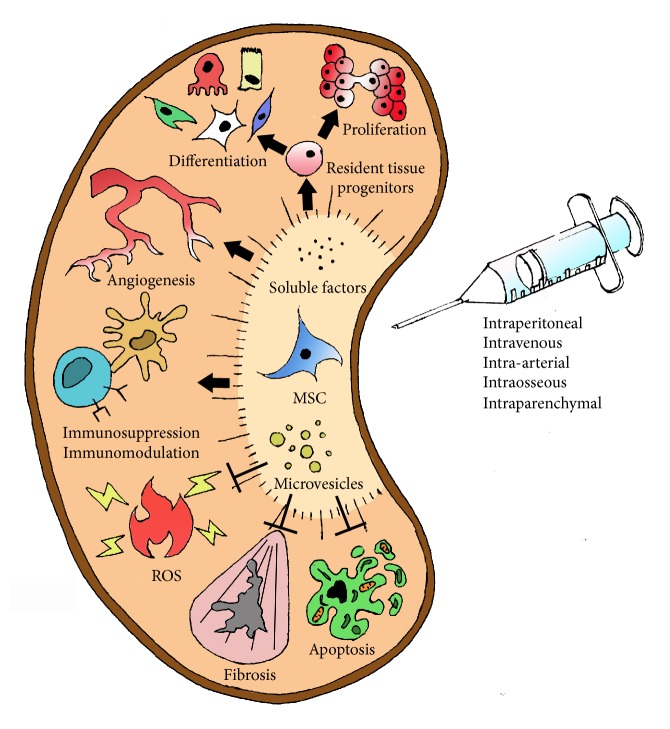
Properties of MSC in kidney diseases. MSC, soluble factors, or microvesicles can be delivered to the kidney via the intraperitoneal, intra-arterial, intravenous, intraparenchymal, or intraosseous route. They exert a series of renoprotective and regenerative actions on the injured tissues through various paracrine mechanisms: antifibrotic and antiapoptotic, proangiogenic, proliferative and differentiative, antioxidative stress, and immunosuppression and immunomodulation of the immune system. ROS: reactive oxygen species. Arrow: enhancement; T-bar: reduction.

MSC form a heterogeneous cell population likely to have a pericytic origin [6]. They can be isolated from several organs besides the bone marrow (bmMSC), such as peripheral blood, connective tissue, skeletal muscle, adipose tissue (adipose-derived MSC, adMSC), dental pulp (dpMSC), umbilical cord wall (ucMSC), umbilical cord blood (cbMSC), amniotic fluid (afMSC), and kidney (kMSC) [18–32]. bmMSC, dpMSC, ucMSC, kMSC, adMSC, and afMSC have all been used in experimental settings to treat various types of renal diseases (Figure 1). While most clinical trials use bmMSC, an increasing number of recent studies have shown that they are difficult to obtain, have ethical issues, and are easily contaminated [33]. Moreover, autologous bmMSC are functionally abnormal in some disorders such as lupus [34, 35], rheumatoid arthritis [36], and systemic sclerosis [37], which may limit their clinical application. As an alternative, ucMSC have been proposed. Umbilical cords fall off after delivery and therefore constitute an easy access to cells, provide less possibilities of contamination, have no ethical concern, and are rich in MSC. Additionally, ucMSC, unlike bmMSC, do not express tumor-associated fibroblast phenotypes and therefore have no opportunity to grow solid tumors [38]. Consequently, several clinical trials on SLE prefer the use of ucMSC (Table 1). Some trials on kidney transplant recipients as well as the one on FSGS and 2 on CKD patients include in their protocol the utilization of adMSC. Adipose tissue is an important source of MSC, with a frequency 100 to 1000 times higher than bmMSC. They also seem to possess a higher potential for angiogenesis or vasculogenesis [39]. Interestingly, a recent study by Bortolotti et al. shows that the therapeutic potential of MSC depends on the source and isolation procedure [40]. In an* in vivo* mouse model of hindlimb ischemia, clinical and histological analysis revealed that bmMSC and adMSC presented different properties. Therefore, while MSC isolated from various tissue have similar characteristics, further characterization would be beneficial for clinical use. Finally, preclinical studies by Melissa Little's group describe the existence of MSC-like cells in the kidney that could support local tissue turnover and/or repair [29]. Their data on variations in the level of epitope presentation and distinct phenotypic signatures between populations provide supporting evidence for a “memory of tissue origin” and suggest the existence of distinct functional roles for MSC-like cells isolated from different tissues. Further investigation will be crucial to the development of future cell therapy approaches to tissue repair as these results hint that finding the best MSC for a particular clinical application will be of paramount importance. In the past few years, MSC have stirred the interest of researchers and clinicians worldwide due to their noteworthy properties (Figure 2).

MSC possess the ability to migrate into damaged tissues in response to combinational signals [17]. This process is called homing and was first reported in leukocyte trafficking [54, 55]. Following injury, MSC preferentially home to inflammatory sites where they migrate across the endothelium and enter the injured tissue bed [56]. Homing occurs through the interaction between signaling molecules released from the damaged tissue, such as chemokines, adhesion molecules and matrix metalloproteinases, and receptors expressed on the MSC surface [57–67].

While initial findings on the therapeutic properties of MSC indicated an important role for homing, engrafting, and differentiation of the cells at the site of injury, numerous additional studies demonstrate a very limited replacement of damaged tissues by transdifferentiation ability and replacement potential [56, 68]. In particular, mechanisms of renal repair observed following ischemia-reperfusion injury do not involve replacement of tubular cells by infused MSC [69–72].

From the first published article in 2000 by Liechty et al., numerous studies have demonstrated the ability of MSC to modulate the immune system [17, 57, 73–75]. MSC express intermediate levels of MHC class I and are negative for the expression of MHC class II and the costimulatory molecules CD40, CD80, and CD86 [76]. While on one hand MSC are protected by the action of natural killer cells and escape recognition of alloreactive T-cells, on the other hand they have a strong immunomodulatory effect and can modulate innate and adaptive compartment through various mechanisms [43, 77–95].

Recent evidence emphasizes the importance of the interactions between the MSC and their environment, as other immunomodulatory properties come into effect in a paracrine/endocrine manner. MSC are able to release dozens of active biological factors that act on local cell dynamics, by decreasing apoptosis, reducing inflammation and fibrosis formation, promoting angiogenesis and recruiting resident progenitor cells, and stimulating mitosis and/or differentiation process [96, 97]. MSC mediate these effects through the secretion of the following.

(i)* Soluble factors* are involved in different processes: (1) immune system signaling like IL-6, IL-8, monocyte chemoattractant protein-1 (MCP-1/CCL2), and TGF-*β*; (2) extracellular matrix remodelers like tissue inhibitor of metalloproteinases 2 (TIMP-2), fibronectin, periostin, collagen, and metalloproteinase inhibitors; (3) growth factor and regulators such as insulin-like factor 1 (IGF-1), hepatocyte growth factor (HGF), and vascular endothelial growth factor (VEGF) [96, 98–104]. These factors can accelerate cellular repair and epithelial proliferation in renal ischemia-reperfusion injury models.

(ii)* Microvesicles* are divisible in shedding vesicles released by membrane budding (particles of 50–200 nm) and exosomes released from intracytoplasmic multivesicular bodies (bilipid membrane vesicles of 50 nm or less). Regardless of their origin, microvesicles are perfect vehicles to deliver mRNA, miRNA, surface receptors, and biologically active molecules like lipids or proteins. These molecules can modulate or reprogram functions of other cells, like enhancing survival and blocking the programmed death system [105–109]. Recently, Ju et al. demonstrated that administration of microvesicles obtained from cultures of human ucMSC in a model of AKI in rat leads to kidney recovery mediated by RNA transfer and synthesis of human HGF [110].

Additionally, recent studies showed positive effect on the kidney structure through fibrosis reduction mediated by MSC. This effect occurs independently of the source of MSC (adMSC, ucMSC, and bmMSC) and injury model (ischemia-reperfusion, IgA nephropathy, and unilateral ureteral obstruction) [111–113].

An additional important property of the MSC is to decrease the severity of organ injury through the reduction of the oxidative stress [114]. Exosomes released by MSC can prevent the accumulation of reactive oxygen species (ROS) or enhance the scavenger activity, and this mechanism was demonstrated in* in vitro* and* in vivo* experiments [41, 105, 115].

## 2. MSC-Based Clinical Trials in Kidney Diseases

The promising results obtained from numerous* in vitro* and* in vivo* experiments using MSC created great enthusiasm in the scientific community, offering new possibilities of cell-based therapies for a wide range of diseases. To date, more than 600 clinical trials conducted worldwide, either completed or ongoing, involve the use of MSC as reported in the US National Institute of Health database (ClinicalTrials.gov). As many as 30 of them use MSC to treat kidney-related diseases, out of which 9 started within the last year (Table 1) [17].

They span a wide range of renal pathologies: acute kidney injury (3 trials), chronic kidney injury (4 trials), focal segmental glomerulosclerosis (1 trial), diabetic kidney disease (1 trial), autoimmune disease (5 trials), and kidney transplantation (16 trials).

### 2.1. Acute Kidney Injury

AKI—previously called acute renal failure—is characterized by the rapid loss of kidney excretory function. Its causes are numerous and can be divided into three categories: prerenal disease such as renal ischemia (from low blood pressure, crush injury, etc.), intrinsic renal disease such as exposure to nephrotoxic substances (antibiotics or contrast agents, e.g.), and systemic disease, or postrenal-like obstruction of the urinary tract. It is typically diagnosed on the basis of characteristic laboratory findings, that is, elevated blood urea nitrogen and creatinine, or decreased urine output, or both [42].

Interesting preclinical results obtained in various mouse models paved the way for the development of novel therapies involving the use of MSC in AKI patients. In fact, no drug is presently available to treat this condition, and the treatment is essentially supportive, including renal replacement therapy whenever necessary. Around 50% of critically ill patients die from AKI, and while most surviving patients completely recover their renal function within weeks, some develop chronic kidney disease (CKD) requiring kidney transplant [116]. However, only three clinical trials have been proposed and their main goal is to investigate the safety and efficacy of allogeneic MSC injection. The first one, (NCT00733876) an exploratory study (phase I), was completed in October 2013 and involved 16 patients. Its aim was to determine the safety and efficacy of bmMSC administration in patients at high risk of developing AKI following on-pump cardiac surgery. The administration route of allogeneic MSC was through the distal thoracic aorta, to avoid cell entrapment in the lungs, which might induce respiratory distress. Study results indicate the absence of specific or serious adverse events during a 6-month follow-up period. Preliminary analysis showed that MSC administration is safe at all tested doses, confers early and late protection of kidney function, and lowers both length of hospital stay and need for readmission [117, 118]. A recently completed phase II trial by Remuzzi's group in oncology patients with cisplatin-induced AKI (NCT01275612) proposes to test the feasibility and safety of systemic infusion of donor* ex vivo* expanded MSC to repair the kidney and improve renal function. A third, larger phase II study (NCT01602328) evaluates kidney recovery following allogenic bmMSC infusion in patients with AKI after undergoing cardiac surgery. No results have been reported so far for these two trials [56, 119].

### 2.2. Chronic Kidney Disease

The number of individuals affected with chronic kidney disease (CKD) is rising worldwide, mainly due to a remarkable increase in atherosclerosis and type 2 diabetes. An estimated 8–16% of the general population has CKD, and its prevalence increases with age to about 30% in people aged over 70 years [120]. CKD is a progressive condition causing significant morbidity and mortality, as patients often develop end-stage renal disease (ESRD) and present an increased risk of cardiovascular disease. It constitutes a significant socioeconomic burden, in particular considering the high cost of renal replacement therapy. Slowing CKD progression is therefore a major health priority [120].

CKD is characterized by reduced renal regenerative capacity. Several* in vivo* studies suggest beneficial regenerative effects of cell-based therapies in animal models of CKD [121]. Administration of both bmMSC and adMSC has demonstrated significant renoprotective effects including reduction of intrarenal inflammatory infiltrate, decreased fibrosis, and glomerulosclerosis. Currently, four phase I clinical trials have been uploaded in the NIH database; all aim to test mainly the safety of using MSC and their efficacy in treating CKD. Two of them propose the use of autologous bmMSC (NCT02166489 and NCT02195323) and two adMSC (NCT02266394 and NCT01840540). These explorative studies are either ongoing or only just completed, and no preliminary result has been provided so far.

### 2.3. Diabetic Kidney Disease

Diabetic kidney disease (DKD)—also called diabetic nephropathy—is a clinical syndrome associated with kidney damage, which can progress to chronic kidney disease. It is the leading cause of ESRD in the industrialized world, accounting for about 40% of new cases in the US and EU. The five-year mortality rate is 39%—a rate comparable to many cancers. The economic cost of DKD and its progression to ESRD represents an astounding 13% of the US healthcare budget. In spite of this enormous social and economic cost, there have been no specific therapies successfully developed for DKD in the past 25 years. The current treatment paradigm relies on early detection, glycemic control, and tight blood pressure management with preferential use of renin-angiotensin system blockade [122]. To address the critical need for a novel therapy for DKD, a controlled phase I/II clinical trial was deposited in October 2015 (NCT02585622), based on the successful preclinical experiments in diabetic mice treated with bmMSC [123]. This study will investigate, primarily, the safety, feasibility, and tolerability and, secondarily, the preliminary efficacy of an allogeneic bmMSC therapy.

### 2.4. Focal Segmental Glomerulosclerosis

Focal segmental glomerulosclerosis (FSGS) is a rare but major cause of ESRD. The rate of recurrence is higher in children compared with adults and in patients submitted to a subsequent kidney transplant. Furthermore, after kidney transplantation, approximately 30–40% of patients with FSGS develop recurrent FSGS. Its incidence is increasing worldwide [124].

In FSGS, glomerular lesions caused by various insults directed to or inherent within the podocyte lead to foot process effacement. The resulting loss of integrity of the glomerular filtration barrier, which regulates permselectivity, causes in turn proteinuria. Traditional pharmacological approaches, consisting of corticosteroids and calcineurin inhibitors, fail to achieve a sustained remission in most patients. Therefore, there is a pressing need to develop alternative therapies for this glomerulopathy [124]. Very few preclinical studies assessing the beneficial effects of MSC infusion in* in vivo* models of FSGS can be found in literature, but all presented promising results, leading to a translation to the clinic [125]. An article by Belingheri et al. from 2013 reports the first allogenic bmMSC treatment in a pediatric recipient of kidney transplantation with a form of FSGS not responding to any conventional and unconventional treatments [126]. Seven, 10, and 14 months following transplant, the patient received bmMSC infusions, divided in three cycles of two infusions (1 × 10^6^ cells/kg/dose) according to the dose most commonly used for graft-versus-host disease (GVHD) treatment. No adverse event was observed, and the patient presented a stable renal function and stabilized proteinuria without the need of further plasmapheresis. In addition, some circulating inflammatory factors decreased and their levels were still low after one year. Recently, a clinical trial (NCT02382874) was opened to evaluate safety and efficacy of intravenous infusion of allogeneic adMSC in 5 refractory FSGS patients. They will be followed up for a year following injection.

### 2.5. Autoimmune Disease: Systemic Lupus Erythematosus

SLE is a chronic autoimmune disease characterized by a wide range of clinical manifestations that can affect many organs in the body, with significant morbidity and mortality. Nephritis remains the most significant manifestation of SLE and standard treatments include high doses of corticosteroids, cyclophosphamides, and other immunosuppressive and biological agents. Most patient outcome improves greatly following therapy, but strong side effects including infection, ovarian failure, and secondary malignancy can worsen the prognosis and lead to patient death [44, 45]. SLE continues to be a therapeutic challenge, and new, more effective, less toxic treatments are needed. While the efficacy of MSC therapy in preclinical models varies and appears to be dependent on the model and the MSC population used, several studies showed that the anti-inflammatory immunomodulatory effects of MSC can be beneficial for SLE patients [46]. Five phase I/II clinical trials can be found on https://clinicaltrials.gov/, examining the safety and therapeutic benefit of MSC therapy in patients with primary or treatment-refractory SLE. Three studies favor the use of ucBMC and two bmMSC. A small pilot study (NCT00698191) involving only four patients with treatment-refractory SLE showed that systemic administration of allogeneic* ex vivo* expanded bmMSC improved renal function and reduced SLE disease activity, such that patients were in disease remission for up to 12 months after treatment [47]. A follow-up study also demonstrated improvement in disease index score, proteinuria, and serological markers in 11 of the 13 patients assessed at 12 months, with the remaining two patients going into relapse [47, 127]. A similar study using ucMSC showed a significant improvement in disease index scoring [51]. None of these trials reported any adverse effects one year from treatment, and therefore potential long-term effects in these studies would require further investigation. A Chinese multicenter study (NCT01741857) involved 40 patients with active and refractory SLE that were injected intravenously with allogenic ucMSC on days 0 and 7 [33]. Safety and remission or relapse were assessed. The overall survival rate was 92.5%, and no transplantation-related adverse events were observed. During the one-year follow-up, 32.5% of patients went into full remission while 27.5% recovered only partially. Additionally, 12.5% went into relapse at 9 months and 16.7% at 12 months. The authors propose to develop a new protocol in which the patients would undergo a second regimen of ucMSC injection after 6 months. Wang et al. then unveiled the putative mechanisms mediating the therapeutic benefit of allogeneic MSC in lupus. In an elegant study, they determined that high levels of interferon-*γ*, produced predominantly by CD8+ T-cells in SLE patients, are a key factor involved in the stimulation of allogeneic ucMSC to produce indolamine 2,3-dioxygenase, which can then inhibit the proliferation of T-cells from SLE patients [128]. Interestingly, a new large-scale clinical trial (NCT02633163) has been uploaded in December 2015 and proposes the injection of either low or high dose of ucMSC or a placebo. This prospective, double-blind, multicenter, controlled study will enroll an estimated 81 treatment-refractory LSE patients and will follow the disease outcome for 1 year. Of note, another two concluded studies have an unknown status (NCT01539902 and NCT00659217).

### 2.6. Kidney Transplantation

Kidney transplant in ESRD patients offers the best chance of survival and improves health-related quality of life compared to remaining on dialysis. Better and more potent immunosuppressive drugs have improved significantly the short-term outcome of the surgery in the last two decades. However, the long-term graft survival rate beyond the first year showed only a small increase [49]. Clinical interest is now focused on reduction of alloimmune injury and immune-suppression-related side effects to optimize preservation of renal function [50, 74, 75]. Given their low immunogenicity and immunoregulatory properties, MSC could potentially be proven beneficial in the context of kidney transplantation. Numerous* in vivo* studies showed that MSC can successfully regulate immune response and support kidney repair [17]. There are currently 16 trials registered on the NIH database, both ongoing and completed, that evaluate the safety and efficacy of MSC infusion following renal transplantation, not in the context of acute clinical rejection [48]. An exploratory study by Perico et al. proposes to test the safety and feasibility of autologous bmMSC injection into two patients with ESRD and undergoing kidney transplant (NCT00752479) [48, 129]. In their experimental protocol, bmMSC were infused intravenously 1 week following surgery and contemporaneously with immunosuppressive drugs. The patients presented a temporary decrease in graft renal function, probably due to the timing of the MSC injection, but displayed a good graft function at one-year follow-up. Additionally, they showed an increased frequency of Treg cells and decreased number of memory CD8+ T-cells. A follow-up study of two patients evaluated the timing of the injection and the necessity of CD25 blockade in the immunosuppressive drug treatment [53, 75]. Therefore, bmMSC were infused one day before kidney transplant. One patient developed acute cellular rejection (ACR) 2 weeks later, due to higher HLA haplotype mismatch, and was treated with steroid pulses. Both patients had excellent graft function during 1-year follow-up. Circulating memory CD8+ T-cells and donor-specific CD8+ T-cell cytolytic response were reduced in MSC-treated patients. CD25 blockade did not affect Treg expansion in MSC-treated patients. In the largest completed study so far (NCT00658073), Tan et al. assessed the benefits of autologous bmMSC injection versus anti-CD25 antibody in ESRD patients that underwent kidney transplant [48, 52]. In patients treated with MSC, they tested a regular dose of calcineurin inhibitors as well as a reduced dose (80% of standard), to prevent organ toxicity. Patient observation at one-year follow-up showed that replacement of CD25 blockade did not affect graft survival. Additionally, MSC treatment conferred faster recovery of renal function, fewer and less severe ACR (7.5% and 7.7% in the MSC group with standard or lower dose of calcineurin inhibitor, versus 21.6% in the CD25 antibody inhibitor group), fewer opportunistic infections, and fewer adverse effects. One-year graft function was comparable in all groups. In a study by Reinders et al. [48, 130], the authors used autologous bmMSC to treat ACR and renal interstitial fibrosis and tubular atrophy in six patients of fully HLA mismatched kidney transplant with subclinical rejection following protocol renal biopsy and/or an increase in interstitial fibrosis/tubular atrophy (NCT00734396). The treatment included full immunosuppressive regiment and intravenous bmMSC injection 6 months after transplant. No adverse effects were noted and two patients showed resolution of tubulitis, while five patients had less donor-specific mononuclear cells, indicating a possible immunomodulatory effect of the MSC. In an ongoing phase II clinical trial by the same group (NCT02057965), 70 renal allograft recipients will receive autologous bmMSC injections or control [131]. Patients in the bmMSC-treated group will receive two doses of bmMSC 7 days apart, 6 and 7 weeks after transplantation in combination with mTOR inhibitors everolimus and glucocorticoid. At the time of the second bmMSC infusion, the calcineurin inhibitor will be reduced to 50% and completely withdrawn 1 week later. Patients in the control group will receive standard immunosuppressive regimen. The end point is the level of fibrosis as well as graft function, occurrence of adverse events, and eventual presence of opportunistic infections in a 6-month follow-up. This study will assert whether bmMSC can be used for tacrolimus withdrawal and whether this strategy leads to preservation of renal structure and function in renal recipients. In a third study, Reinders et al. will assess the safety and feasibility of using allogenic bmMSC in 10 renal transplant recipients (NCT02387151) [132]. Indeed, allogenic bmMSC offer the advantage of immediate availability for clinical use. This is of major importance for indications where instant treatment is needed, for example, allograft rejection or calcineurin inhibitor toxicity. Although rare previously published studies showed no adverse reactions, allogeneic MSC could possibly elicit an antidonor immune response, which may increase the incidence of rejection and affect the allograft survival in the long term. Patients will receive two doses of bmMSC intravenously, at 25 and 26 weeks after transplantation, when immune suppression levels are reduced. The primary end point of this study is graft loss, while the secondary includes comparison of fibrosis in renal biopsy,* de novo* HLA antibody development and extensive immune monitoring, renal function, and opportunistic infections. An unregistered small clinical trial already assessed the safety and efficacy of autologous bmMSC transplantation in four patients that underwent living-donor renal transplantation and the effect on the immunophenotype and functionality of peripheral T lymphocytes following transplantation [133]. All patients developed no immediate or delayed adverse effects at the 6-month follow-up. Graft function was good and protocol biopsies at 1 and 3 months did not reveal any abnormality. Compared to baseline, there was an increase in Treg cells and reduction in CD4+ T-cell proliferation which led to the conclusion that autologous bmMSC are beneficial in renal transplantation. However, larger randomized trials studies are needed to confirm these findings and evaluate whether this will have any impact on immunosuppressive therapy. Another four studies not registered on https://clinicaltrials.gov/ reported interesting result. In the first one, Vanikar et al. [48, 134] evaluated, in 100 renal allograft recipients for ESRD, the donor hyporesponsiveness to donor adMSC combined with hematopoietic stem cell transplantation (HSCT) versus HSCT alone, under nonmyeloablative conditioning. The adMSC group showed improved graft survival and sustained chimerism levels compared to the control group in the 18-month follow-up period. In a subsequent large-scale trial [135] involving 916 patients undergoing living-donor kidney transplantation, the authors tested the induction of hyporesponsiveness protocol with donor-specific adMSC versus controls receiving conventional triple immunosuppression regimen. The preliminary analysis of the results obtained in this study shows that adMSC transplantation is effective in minimization of immunosuppression in kidney transplant, resulting in good graft function and patient and graft survival at 4 years [135]. However, this study lacks a control group of patients receiving nonmyeloablative conditioning with no adMSC injection. In a small clinical trial, seven HLA cross-matched living-donor kidney transplant recipients were given simultaneously donor bmMSC injection into the iliac bone [48, 136]. Neither adverse event nor graft failure was observed, but biopsy-proven ACR were detected in three recipients during the follow-up period and required steroid pulse therapy. Donor-specific lymphocyte or T-cell proliferation and Treg priming responses were occasionally observed. This study supports the feasibility of the treatment, but additional studies should ascertain the impact of allogenic bmMSC injection on graft outcome on a larger cohort of patients with control groups [48]. Peng et al. tested the safety and efficacy of donor bmMSC infusion through the renal artery combined with reduced calcineurin inhibitor treatment in living-donor kidney transplant recipients compared with control patients that received the standard immunosuppressive regimen [48, 137]. Patients in the experimental group maintained a stable graft function during the one-year follow-up period and displayed higher number of peripheral B-memory cells at 3 months. No chimerism was detectable at 3 months. These preliminary data suggest that the use of bmMSC could reveal itself beneficial in renal transplantation by reducing the dosage of conventional immunosuppressive drug that is required to maintain long-term graft survival and function. Another 10 phase I/II clinical trials are currently ongoing and have not reported any results yet. Only one completed study (NCT00659620) has an unknown status and did not present any publication. It is clear that MSC-based therapy in kidney transplantation is in its infancy, and no real evidence of its benefits for the patient has been shown so far.

It is noteworthy that one registered clinical trial aims to compare the use of autologous and allogenic bmMSC treatment in kidney transplantation patients and will help to elucidate the effect of the bmMSC on the T-cell repertoire of the recipients (NCT02409940). Presently, both autologous and allogenic MSC are used in cell therapy, and some questions remain regarding which cell type leads to the best disease outcome. The use of autologous MSC is not always preferred nor possible because patients can present cells with reduced qualities or quantities [138]. For example, diabetes negatively impacts MSC, as it lowers the angiogenic capacity of the cells and therefore their therapeutic potential [139]. Autologous bmMSC in patients with certain immunologic disorders are abnormal and therefore less desirable in clinical trials [34–37]. Additionally, certain genetic disorder may impede the use of autologous MSC. In a study on multiple myeloma patients, the authors have found, based on analysis of cellular receptors, growth factors, and cytokine expression, that myeloma bmMSC are phenotypically and functionally distinguishable from normal donor MSC [140]. In patients with hematological malignancies, chemotherapeutic treatments damage qualities and lower numbers of MSC [141]. Consequently, allogenic MSC are often used in clinical trials. As previously stated, an important property of MSC is the absence of MHC class II molecules as well as costimulatory molecules on their cell surface, allowing them to evade allogeneic rejection. Additionally, they offer several advantages over autologous MSC: donors can be thoroughly screened and tested for MSC, and a single donor can serve for multiple recipient, becoming some kind of “qualified donor,” taking into consideration all of his characteristics.

Over the past several years, the discrepancy between the number of wait-listed patients and the number of kidneys from brain-dead donors has been increasing steadily, leading to a shortage of organs and resulting in an extension of the criteria for kidney donors, including non-heart-beating donors (NHBD) [142]. However, kidneys from NHBD suffer damage during the period of warm ischemia associated with the cardiac death [143]. The most common consequence of the use of these suboptimal kidneys is the increase in delayed graft function, the clinical pendant of AKI [142]. As previously discussed in Acute Kidney Injury, a vast body of preclinical evidence highlights the benefits of using MSC infusion to protect and enhance the repair process in ischemic kidneys, and three clinical trials are already ongoing [144]. In fact, these studies form the rational of using MSC in the context of kidney transplantation from NHBD and should allow extending even further the use of organs from marginal donors.

## 3. Conclusions

MSC form a population of well-characterized, easily obtainable cells with therapeutic properties effective in numerous experimental models of kidney diseases. The underlying mechanisms of action of the MSC have been extensively described and consist essentially in immunomodulatory and paracrine effects. However, the translation of preclinical studies into robust, effective, and safe patient therapies remains limited. The many clinical trials that have been conducted and completed will undoubtedly provide further insight into safety, feasibility, and efficacy of MSC-based therapy in renal pathologies. The preliminary results available still lack long-term follow-up data and the absence of consensus between therapeutic protocols, in particular in terms of MSC preparation, donor characteristics, and concomitant immunosuppressive treatment in kidney transplant recipients, is noteworthy. As a broad range of approaches have been developed, a careful selection of the best one will have to be made in the future in an effort to reach a certain harmonization in clinical practices [17, 48, 145]. Recent studies suggest the possibility of potentiating the intrinsic reparative capacity of MSC through preconditioning or genetic modification [138, 146–148]. Once fully tested, enhanced MSC could become an important ne tool for current as well as unexplored therapeutic fields.

## Figures and Tables

**Table 1:** Current clinical trials conducted worldwide using MSC to treat kidney diseases, from the US National Institute of Health database (ClinicalTrials.gov).

NCT number/references	Title	Trial centers	Phase	Conditions	Primary end point	Secondary endpoint	Follow-up period	Enrollment (planned)	Type of MSC	Cell regimen	Therapy (control/placebo)	Start and completion date/status
*Acute kidney injury*												
NCT01275612	Mesenchymal stem cells in cisplatin-induced acute renal failure in patients with solid organ cancers	Bergamo, Italy	I	Cisplatin-induced AKI	Rate of renal function loss (sCr)	NGAL, NAG	1 month	9	Allogeneic bmMSC	Single i.v. infusion Experimental 1: 1 × 10^6^ MSC/kg Experimental 2: 2 × 10^6^ MSC/kg Experimental 3: 5 × 10^6^ MSC/kg	Single group assignment	Nov 2010–Mar 2016; recruiting
NCT01602328	A study to evaluate the safety and efficacy of AC607 for the treatment of kidney injury in cardiac surgery subjects	AlloCure Inc., Burlington, Massachusetts, USA	II	Postcardiac surgery AKI	Time to kidney recovery (sCr)	All-cause mortality or dialysis	36 months	156	Allogeneic AC607 bmMSC	Single i.v. infusion Active comparator: 1 × 10^6^ MSC/kg Placebo comparator: vehicle only	Randomized, parallel assignment, double-blind, placebo-controlled	Jun 2012–Aug 2014; completed
NCT00733876 [41, 42]	Allogeneic multipotent stromal cell treatment for acute kidney injury following cardiac surgery	AlloCure Inc., Burlington, Massachusetts, USA	I	Postoperative AKI (patients who require on-pump cardiac surgery)	Absence of MSC-specific adverse or serious adverse events		36 months	15	Allogeneic bmMSC	Experimental: dose-escalating intra-aortic infusion	Nonrandomized, single group assignment	Aug 2008–Oct 2013; completed

*Chronic kidney disease*												
NCT02166489	Mesenchymal stem cells transplantation in patients with chronic renal failure due to polycystic kidney disease	Tehran, Islamic Republic of Iran	I	Chronic renal failure due to autosomal dominant polycystic kidney disease (ADPKD)	Probability of mass formation in patients with PKD	Renal function (GFR)	18 months	6	Autologous bmMSC	Experimental: single i.v. infusion 2 × 10^6^ MSC/kg	Single group assignment	Mar 2014–Jan 2016; completed
NCT02266394	Hypoxia and inflammatory injury in human renovascular hypertension	Birmingham, Alabama; Rochester, Minnesota; Jackson, Mississippi, United States	I	Renal artery stenosis, ischemic nephropathy, renovascular disease, chronic kidney disease in human renovascular hypertension	Renal function, safety of MSC infusion	Decrease in kidney inflammation	36 months	42	Autologous adMSC	Active comparator 1: single i.a. infusion Active comparator 2: single i.a. infusion and after i.a. stent placement	Nonrandomized, parallel assignment	Oct 2014–Mar 2019; recruiting
NCT01840540	MSC for occlusive disease of the kidney	Rochester, Minnesota, United States	I	Atherosclerotic renal artery stenosis, ischemic nephropathy, renovascular hypertension	Renal blood flow (CT), renal function (GFR)	Blood pressure levels (oscillometric measurement)	24 months	6	Autologous adMSC	Experimental: single i.a. infusion	Single group assignment	Apr 2013–Apr 2017; ongoing
NCT02195323	Autologous bone marrow derived mesenchymal stromal cells (bmMSC) in patients with chronic kidney disease (CKD)	Tehran, Islamic Republic of Iran	I	Chronic kidney disease	Mass formation, renal function (sCr)	GFR	18 months	7	Autologous bmMSC	Experimental: single i.v. infusion 2 × 10^6^ MSC/kg	Single group assignment	Apr 2014–Jan 2016; completed

*Focal segmental glomerular sclerosis*												
NCT02382874	Allogenic adMSC transplantation in idiopathic nephrotic syndrome (focal segmental glomerulosclerosis)	Tehran, Islamic Republic of Iran	I	Focal segmental glomerulosclerosis	Renal function (sCr, proteinuria)	Renal function (sCr, urea, GFR), increase in anti-inflammatory factors (sIL-2, I-10), increase in Treg	12 months	5	Allogeneic adMSC	Experimental: single i.v. injection	Single group assignment	May 2015–Oct 2017; recruiting

*Diabetic kidney disease*												
NCT02585622	Novel stromal cell therapy for diabetic kidney disease (NEPHSTROM)	Galway, Ireland; Bergamo, Italy; Belfast, United Kingdom; Birmingham, United Kingdom	I/II	Diabetic kidney disease	Number of adverse events	GFR, UAE	24 months	48	Allogeneic bmMSC	Experimental: MSC i.v. infusion 3 doses 80, 160, 240 × 10^6^ MSC Placebo comparator: only vehicle	Randomized, parallel assignment, double-blind, placebo-controlled	May 2016–Apr 2019; not yet recruiting

*Autoimmune disease*												
NCT00698191 [43–45]	Mesenchymal stem cells transplantation for refractory systemic lupus erythematosus	Nanjing, Jiangsu, China	I/II	Refractory systemic lupus erythematosus	Systemic lupus erythematosus disease activity index (SLEDAI), lupus serology (ANA, dsDNA, C3, C4), renal function (GFR, BUN, urinalysis)	Percentage of systemic T regulatory population	24 months	20	Allogeneic bmMSC	Experimental: pretreatment with cyclophosphamide then transplantation i.v. with 10^6^ cells/kg MSC	Nonrandomized, single group assignment	Mar 2007–Dec 2012; unknown, not verified recently
NCT01539902	Phase 2 study of human umbilical cord derived mesenchymal stem cell for the treatment of lupus nephritis	Kunming, Yunnan, China	II	Lupus nephritis	Efficacy and safety (renal function, urinary RBC, proteinuria)		6 months	25	Allogeneic ucMSC	Experimental: MSC i.v. infusion Placebo comparator: cyclophosphamide	Randomized, double-blind, parallel group, placebo controlled	Feb 2012–May 2013; unknown, not verified recently
NCT02633163	A controlled trial of allogeneic mesenchymal stem cells for the treatment of refractory lupus	Los Angeles, California; Atlanta, Georgia; Chicago, Illinois; Rochester, New York; Chapel Hill, North Carolina; Charleston, South Carolina, United States	II	Systemic lupus erythematosus	Clinical response defined by the SLE responder index	Change in SLEDAI score, renal and nonrenal organ system flares	12 months	81	Allogeneic ucMSC	Experimental 1: single MSC i.v. infusion 1 × 10^6^ MSC Experimental 2: single MSC i.v. infusion 5 × 10^6^ MSC Placebo comparator: only vehicle	Randomized, double-blind, placebo controlled	Jul 2016–Jun 2021; not yet recruiting
NCT01741857 [46, 47]	Umbilical cord derived mesenchymal stem cells transplantation for active and refractory systemic lupus erythematosus	Nanjing, Jiangsu, China	I/II	Systemic lupus erythematosus	BILAG score	Lupus serology (Alb, ANA, dsDNA, C3, C4), renal function (GFR, BUN, urinalysis)	12 months	40	Allogeneic ucMSC	Experimental: MSC transplantation	Single group assignment	Jan 2012–Dec 2013; unknown, not verified recently
NCT00659217	Effect of mesenchymal stem cell transplantation for lupus nephritis	Fuzhou, Fujian, China	I/II	Lupus nephritis	Number of achieved and maintained remissions	Patient survival, sCr and proteinuria, SLE disease activity index, serology (ANA, dsDNA), complement (C3 and C4)	12 months	20	Autologous MSC	Experimental 1: prednisone administration Active comparator 2: MSC infusion	Single group assignment	May 2008–May 2010; unknown, not verified recently

*Kidney transplantation*												
NCT00659620	Mesenchymal stem cell transplantation in the treatment of chronic allograft nephropathy	Fuzhou, Fujian, China	I/II	Kidney transplant, chronic allograft nephropathy	Renal function (sCr and Cr clearance rate)	Patient and graft survival, the proportion of renal biopsy, the incidence of infectious complications Incidence of adverse events associated with MSC and immunosuppression	12 months	20	Allogeneic MSC	Experimental 1: MSC infusion and full immunosuppressive therapy Placebo comparator: full immunosuppressive therapy	Randomized, placebo-controlled	May 2008–May 2010; unknown, not verified recently
NCT02409940	To elucidate the effect of mesenchymal stem cells on the T-cell repertoire of the kidney transplant patients	Chandigarh, India	I	Renal transplant rejection	T-cell expansion, renal function (sCr)	T-cells proliferation changes, regulatory T-cells changes, memory T-cells changes, B-cells changes, cytokine profile change	24 months	30	Allogeneic/ autologous MSC	Experimental: two doses of autologous MSC infusion, one day before transplant and 30 days after transplant Active comparator: two doses allogeneic MSC infusion one day before transplant and 30 days after transplant Placebo comparator: only vehicle	Randomized, parallel assignment	Sep 2013–Dec 2016; recruiting
NCT02561767	Effect of bmMSC in DCD kidney transplantation	Guangdong, China	I/II	Kidney transplantation, acute kidney tubular necrosis	Renal function (estimated GFR)	Incidence of slow graft function, incidence of delayed graft function, proportion of normal renal function recovery, time to renal function recovery, patient survival, renal graft survival, incidence of acute rejection, severe adverse events	12 months	120	Allogeneic bmMSC	Experimental: four doses of MSC 1 × 10^6^ i.v. infusion at days 0, 7, 14, 21 after renal artery reperfusion and induction therapy Placebo comparator: only vehicle at days 0, 7, 14, 21 and induction therapy	Randomized, parallel assignment, single-blind, placebo-controlled	Oct 2015–Oct 2017; completed
NCT01429038	Mesenchymal stem cells after renal or liver transplantation	Liège, Belgium	I/II	Kidney failure	Safety (MSC infusion toxicity), incidence of infections and cancers	Patient and graft survivals, feasibility and safety, effects of MSC on graft function, rejection rates, recipient's immune function, development of anti-MSC donor HLA antibodies	24 months	40	Allogeneic bmMSC	Experimental: single MSC infusion 1, 5–3, 0 × 10^6^	Nonrandomized, parallel assignment	Feb 2012–Feb 2017; recruiting
NCT00658073 [48]	Induction therapy with autologous mesenchymal stem cells for kidney allografts	Fuzhou, Fujian, China		Renal transplant rejection	Incidence of acute rejection and early renal function recovery	Patient and graft survival and prevalence of adverse events	12 months	165	Autologous bmMSC	Active comparator 1: two infusions of MSC, one at releasing renal artery clamp and one two weeks after transplantation and regular immunosuppressive agents Active comparator 2: two infusions of MSC, one at releasing renal artery clamp and one two weeks after transplantation and immunosuppressive agents with 80% less calcineurin inhibitor Active comparator 3: anti-interleukin 2 receptor antibody and regular immunosuppressive agents	Randomized, parallel assignment	Mar 2008–Oct 2010; completed
NCT02563366	Effect of bmMSC on early graft function recovery after DCD kidney transplant	Guangzhou, Guangdong, China	I/II	Kidney transplantation, acute kidney tubular necrosis	Renal function (estimated GFR)	Proportion of normal renal function recovery, time to renal function recovery, acute rejection rate, patient and graft survival rate, incidence of severe adverse events	12 months	120	Allogeneic bmMSC	Experimental: four i.v. administration doses of MSC 1 × 10^6^ every week Placebo comparator: only vehicle every week	Randomized, parallel assignment, single-blind	Nov 2015–Dec 2017; not yet recruiting
NCT02490020	A perspective multicenter controlled study on application of mesenchymal stem cell (MSC) to prevent rejection after renal transplantation by donation after cardiac death	Guangzhou, Guangdong, China	I	Disorder related to renal transplantation, renal transplant rejection	Safety (Incident rates of BPAR and DGF)		12 months	260	bmMSC	Experimental 1: routine treatment protocol plus MSC i.v. 2 × 10^6^/Kg 48 hours before operation Placebo comparator 1: routine treatment protocol Experimental 2: routine treatment protocol plus MSC i.v. 2 × 10^6^/Kg plus MSC i.a. 2 × 10^6^/Kg 48 hours before operation Placebo comparator 2: routine treatment protocol Experimental 3: routine CMR treatment protocol plus MSC i.v. 2 × 10^6^/Kg at days 1, 7 Placebo comparator 3: routine CMR treatment protocol Experimental 4: routine AMR treatment protocol plus MSC i.v. 2 × 10^6^/Kg at days 1, 7 Placebo comparator 4: routine AMR treatment protocol	Randomized, parallel assignment, single-blind	Jan 2016–Dec 2018; enrolling by invitation
NCT00752479 [49, 50]	Mesenchymal stem cells under Basiliximab/low dose RATG to induce renal transplant tolerance	Bergamo, Italy		Kidney transplant	Inhibition of memory T-cell response and/or naive T-cell response, Induction of donor-reactive T-cell anergy and the appearance in the peripheral blood of regulatory T-cells	Safety of MSC infusion, graft function, graft rejection	12 months	4	Syngeneic bmMSC	Experimental: MSC infusion 2 × 10^6^/Kg at the time of kidney transplant plus induction and maintenance therapy Active Comparator: immunosuppressive therapy plus induction and maintenance therapy	Randomized, parallel assignment	May 2008–Dec 2013; completed
NCT02563340	Effect of bmMSC on chronic AMR after kidney transplantation	Guangzhou, Guangdong, China	I/II	Kidney transplant	Renal function (estimated GFR)	Patient survival rate, graft survival rate, DSA level, pathological manifestation (Banff 2013 criteria), severe adverse events	12 months	60	Allogeneic bmMSC	Experimental: four i.v. MSC infusions 1 × 10^6^ plus desensitization therapy Active comparator: desensitization therapy	Nonrandomized, parallel assignment, single-blind	Nov 2015–Nov 2017; not yet recruiting
NCT02492490	Effect of SVF derived MSC in DCD renal transplantation	Fuzhou, Fujian, China	I/II	Kidney transplant	Safety (incidence of DGF: 3-month reduction of CNI)	Renal function (eGFR, proteinuria), incidence of acute rejection, allograft survival, SAE, nonhematologic toxicities	12 months	120	Autologous adMSC	Experimental: four i.v. MSC infusions during kidney transplant operation and at days 7, 14, 21 Active comparator: Basiliximab administration	Randomized, parallel assignment	Dec 2014–Nov 2016; recruiting
NCT00734396 [51]	Mesenchymal stem cells and subclinical rejection	Leiden, Netherlands	I/II	Kidney transplant	Rate of (serious) adverse events, feasibility (number of expanded MSC in relation to the amount of BM collected)	Acute rejection, renal cortical matrix accumulation, immunologic response evaluation	24 months	15	Autologous bmMSC	Experimental: two i.v. MSC infusions 1-2 × 10^6^/Kg	Nonrandomized, single group assignment	Feb 2009–Dec 2012; completed
NCT02492308	Induction with SVF derived MSC in living-related kidney transplantation	Fuzhou, Fujian, China	I/II	Living-relative kidney transplantation	Effects on dosage of immunosuppressant	Renal function (eGFR, proteinuria), incidence of acute rejection, allograft survival, infection adverse event, nonhematologic toxicities, hematologic toxicities, incidence of delayed graft function	12 months	120	Autologous adMSC	Experimental: four i.v. MSC infusions during kidney transplant operation and at days 7, 14, 21 Active comparator: Basiliximab administration	Randomized, parallel assignment	Dec 2014–Dec 2017; recruiting
NCT02387151 [52]	Allogeneic mesenchymal stromal cell therapy in renal transplant recipients	Leiden, Netherlands	I	Rejection, graft loss	Biopsy proven acute rejection/graft loss	Comparison of fibrosis by quantitative Sirius Red scoring, serious adverse events, renal function measured by eGFR (MDRD formula) and iohexol clearance, CMV, BK infection (viremia, disease, and syndrome; and subtypes of BK viremia) and other opportunistic infections, development of *de novo* DSA and immunological responses	12 months	10	Allogeneic bmMSC	Experimental: two i.v. MSC infusions 1-2 × 10^6^/Kg at weeks 25, 26 after transplantation	Single group assignment	Mar 2015–Mar 2017; recruiting
NCT02012153	Mesenchymal stromal cells in kidney transplant recipients	Bergamo, Italy	I	Kidney transplant rejection	Naive and memory T-cell count (CD45RA/CD45RO), T-cell function (ELISPOT assay), number of adverse events, regulatory T-cell count, urinary FOXP3 mRNA expression (RT qPCR)		12 months	6	Autologous bmMSC	Experimental: single i.v. MSC infusion 2 × 10^6^/Kg the day before the kidney transplant procedure	Single group assignment	Dec 2013–Mar 2018; recruiting
NCT02565459	MSC and kidney transplant tolerance	Bergamo, Italy	I	Kidney transplant	Number of adverse events, T-cell function, urinary FOXP3 mRNA expression (RT qPCR), naive and memory T-cell count (CD45RA/CD45RO), regulatory T-cell count		12 months	22	Allogeneic bmMSC	Experimental: single i.v. MSC infusion 1-2 × 10^6^/Kg Placebo comparator: no intervention	Randomized, parallel assignment	Sep 2015–Dec 2021; recruiting
NCT02057965 [53]	Mesenchymal stromal cell therapy in renal recipients	Leiden, Netherlands	II	Renal transplant rejection, fibrosis	Histology (fibrosis evaluation by Sirius Red)	Renal function and proteinuria, number of participants with CMV and BK infection and other opportunistic infections between groups, number of participants with adverse events, composite, end point efficacy failure, presence of donor specific antibodies and immunologic monitoring	6 months	70	Autologous bmMSC	Experimental: three i.v. MSC infusions 1-2 × 10^6^/Kg 7 days apart, 6 and 7 weeks after transplantation plus Everolimus administration Placebo comparator: tacrolimus plus Everolimus administration	Randomized, parallel assignment	Mar 2014–Mar 2017; recruiting

ADPKD: autosomal dominant polycystic kidney disease; ALB: albumin; ANA: antinuclear antibodies; BILAG: British Isles Lupus Assessment Group; BPAR: biopsy-proven acute rejection; BUN: blood urea nitrogen; CMV: cytomegalovirus; CNI: calcineurin inhibitor; DGF: delayed graft function; DSA: donor-specific antibody; ELISPOT: enzyme-linked immunospot; GFR: glomerular filtration rate; HLA: human leukocyte antigen; i.a.: intra-arterial; i.v.: intravenous; MDRD: modification of diet in renal disease; NAG: N-acetyl-p-D glucosaminidase enzyme; NGAL: neutrophil gelatinase-associated lipocalin; RBC: red blood cells; SAE: severe adverse effects; sCr: serum creatinine; SLE: systemic lupus erythematosus; SLEDAI: systemic lupus erythematosus disease activity index; UAE: urinary albumin excretion.
